# Current status of periodontitis and its association with tobacco use amongst adult population of Sunsari district, in Nepal

**DOI:** 10.1186/s12903-021-01416-3

**Published:** 2021-02-12

**Authors:** K. Goel, S. Sharma, D. D. Baral, S. K. Agrawal

**Affiliations:** 1 Department of Periodontology and Oral Implantology, College of Dental Surgery, B.P Koirala Institute of Health and Sciences, Dharan, 56700 Nepal; 2 School of Public Health and Community Medicine, B.P Koirala Institute of Health and Sciences, Dharan, 56700 Nepal; 3 Department of Public Health Dentistry, College of Dental Surgery, B.P Koirala Institute of Health and Sciences, Dharan, 56700 Nepal

**Keywords:** Periodontitis, Prevalence, Smoking, Smokeless tobacco

## Abstract

**Background:**

Tobacco products are considered significant, but preventable factors related to initiation and progression of periodontal diseases. We assessed the prevalence of periodontitis and evaluated its association with tobacco use and other factors amongst the adult population of Sunsari district in eastern Nepal.

**Methods:**

A community-based, cross-sectional study was conducted in rural municipalities in the province one of eastern Nepal. A total of 440 adults were interviewed with a set of a pre-tested semi-structured questionnaire. Data on social demographics, adverse oral habits followed by periodontal clinical examination were recorded. Prevalence of periodontitis was assessed by a case definition provided by CDC-AAP. Univariate and multivariate logistic regression analysis was done to measure the association between tobacco use and other factors with periodontitis.

**Results:**

The overall prevalence of periodontitis was found to be 71.6%. Majority (85.4%) of tobacco users had periodontitis and they were significantly associated with the disease and its severity. The study identified age groups, 45–65 years (AOR = 7.58, 95% CI 3.93–14.61), plaque accumulation (AOR = 1.01, 95% CI 1.00–1.02), smoking (AOR = 3.14, 95% CI 1.36–7.27), khaini users (smokeless tobacco, AOR = 2.27, 95% CI 1.12–4.61) and teeth loss (AOR = 2.02, 95% CI 1.21–3.38) as the significant factors associated with periodontitis.

**Conclusion:**

The prevalence of periodontitis is high in the surveyed rural adult population. Cigarette smoking along with the use of smokeless tobacco in the form of khaini were identified as significant factors associated with periodontitis.

**Supplementary Information:**

The online version contains supplementary material available at 10.1186/s12903-021-01416-3.

## Background

Periodontal diseases are a result of a disruption in the host microbial interaction, and are known to be one of the major causes of tooth loss [1]. Overall, this disease affects about 20–50% of the global population [2] and in its severe form, ranks sixth among the most prevalent disorders [3]. Although dental plaque-associated microorganism are the primary etiologic agent, several other factors such as genetic, systemic, immunological, environmental and behavioral factors play an important role in determining the susceptibility of individuals to periodontal diseases [4, 5].

Among the environmental factors, tobacco smoking is considered one of the true risk factors and is known to be independently related to periodontal destruction [6]. The common forms of tobacco smoking are cigarette, beedi, chutta and hooka, with cigarettes being the main product smoked [7]. More than seven thousand toxins are present in tobacco smoke [8] including, carcinogens and addictive psycho-active substances like nicotine, which are detrimental to general health and also a major public health concern [9]. In addition, the use of smokeless tobacco (SLT) as an alternative tobacco product to cigarette smoking is gradually becoming popular. An estimated 346 million people in the world use SLT products and the prevalence of use is relatively high in Southeast Asian region accounting for nearly 86% of the global users [10].

SLT is consumed un-burnt and exists in numerous forms across the globe with various applications, e.g. in the USA, SLT is available in the form of chewing and snuff (moist and dry) and in Sweden it is available as snus [11]. In South East Asian countries, most commonly available and used SLT products is “khaini” (powdered tobacco/leaves with slaked lime paste) that is placed in the mouth for use or held between the gum and cheeks for a varied amount of time. Other products that are ingested through the oral tissues, chewed or swallowed [12] are betel quid with or without tobacco like zarda (boiled tobacco leaves with water and slaked lime) and gutkha (areca nut with added tobacco, slaked lime and catechu) [11, 13]. Unlike tobacco smoke that is a risk for overall periodontitis, reports suggest smokeless tobacco products have a greater clinical attachment loss (CAL) only near the area where the products are placed in the mouth [14]. However, conclusion vary when the loss of interproximal bone is discussed. Few studies corroborate the association between SLT use and bone loss [15] but it is not in agreement with others [14, 16]. SLT use and generalized periodontitis is also, a debatable issue as the use is not necessarily associated with overall periodontitis.

Early evidence has given an indication that the Nepalese population is highly susceptible to periodontitis and other oral health related problems [17]. The rising cost of dental services and lack of proper oral hygiene practices contributes to poor oral health status in Nepal. In addition, smoking habits as well as consumption of smokeless tobacco which are common and prevalent in Nepal [18], further contribute to the oral health related problems. Hence, identification of socio-demographic factors, habits and disease prevalence becomes crucial to take action, promote and implement oral health interventions in rural and urban areas. The aims of this study is therefore, to assess the prevalence of periodontitis and to evaluate its association with tobacco use and other factors among the adult population of eastern Nepal.

## Methods

**Fig. 1:**
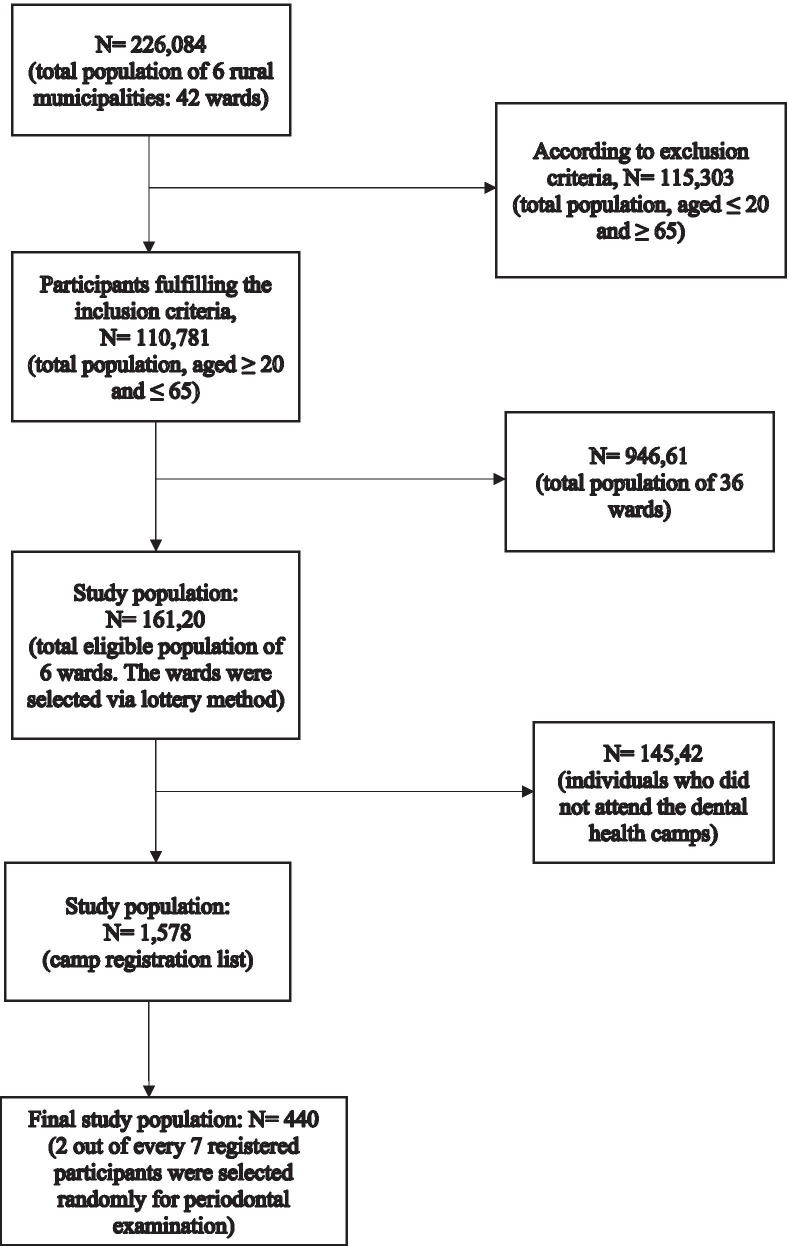
Flow diagram of participants

A cross-sectional study was carried over a period from April 2018 to July 2019 with the approval of the Institutional Review Committee of B.P Koirala Institute of Health Sciences, Dharan, Nepal. The principles of the Declaration of Helsinki were followed during this study. The study obtained written consent from each participant after explaining the objectives, and use of the study. The population involved in this study were inhabitants from the rural area of Sunsari district, in the eastern region of Nepal. Sunsari district comprises of twelve, both rural and urban municipalities. Dental health camps were organized in different wards of six rural municipalities (Koshi, Gadhi, Barju, Bhokraha, Harinagara, Dewanganj) by Department of Public Health Dentistry. The study selected one ward, in each of the six rural municipalities, based on a lottery method. The total eligible population of these wards was approximately 16,120 (estimated population between the age of 20–65 years) [19]. Approximately 1578 inhabitants, who attended the dental health camps were registered in the camp register list, and two out of every seven registered participants were selected in a random manner and examined. Among them a total of 440 participants who met the inclusion criteria were interviewed and enrolled in the study (Fig. 1).

### Criteria for selection: inclusion criteria

Patients between 20 and 65 years old, tobacco users who were currently consuming tobacco in the form of smoking or smokeless tobacco, non-tobacco users who had never used tobacco in any form (smoke or smokeless tobacco), and patient who consented for clinical examination and answered the comprehensive questionnaire.

### Exclusion criteria

Former smokers, patients who actively consume alcohol, patients suffering from known systemic illness, pregnant and lactating females.

### Method of data collection

A set of pre-tested semi- structured questionnaire was prepared and face-to-face interviews in local language were conducted to record the data on social demographics, adverse oral habits and oral hygiene status. The questionnaire is available as the Additional file 1. Age groups were categorized into three categories, as “20–34”, “35–44”, “45–65” years and Body Mass Index (BMI) was calculated as body weight (kg) divided by height (m^2^). Socio-economic status was assessed and categorized into upper, lower middle and lower class [20]. To ensure objectivity, direct questions were asked to the participants regarding their use of tobacco. Current smokers were defined as subjects smoking more than five cigarettes per day for the past 2 years or more, and subjects consuming smokeless tobacco on a daily basis for the past 2 years or more [21].

The SLT users were dichotomized as participants who consumed khaini and those who chewed SLT (gutkha, betel quid with tobacco, zarda). Intraorally, Plaque Index (PLI) was recorded as the presence or absence of visible plaque [22]. Bleeding Point Index (BPI) was used to examine presence or absence of bleeding on probing [23]. Simplified Oral Hygiene Index (OHI-S) was recorded according to Greene and Vermillion, 1964 [24]. A periodontal probe, UNC-15 (University of North Carolina-15, Hu-Friedy, Chicago, IL) was used for all periodontal recordings. To avoid inter-observer variation, a single experienced periodontist (K.G) examined all subjects and the dental hygienist (K.T) was trained by the researcher to fill the questionnaire form. The kappa statistics was assessed among the forty-four participants who were not enrolled in the study. The participants were re-examined after one week of first examination and the value averaged 0.8 for intra-examiner reliability. The prevalence of periodontitis in this study was estimated based on the case- definition given by CDC-AAP in 2012 [25]. Absence of periodontitis was defined as, no indication of mild, moderate, or severe periodontitis. Mild periodontitis: two or more interproximal sites with CAL of ≥ 3 mm, and ≥ 2 interproximal sites with Probing Depth (PD) ≥ 4 mm (not on same tooth) or one site with PD ≥ 5 mm. Moderate periodontitis: two or more interproximal sites with CAL of ≥ 4 mm (not on same tooth), or ≥ 2 sites with PD ≥ 5 mm (not on same tooth). Severe periodontitis: two or more interproximal sites with CAL of ≥ 6 mm (not on same tooth) and ≥ 1 site with PD ≥ 5 mm [25]. PD was measured to the nearest millimeter as the distance from the gingival margin to the bottom of the periodontal sulcus/pocket (cut-offs at ≥ 4 mm and ≥ 5 mm). CAL was computed from the Cemento-Enamel Junction (CEJ) to the base of pocket/sulcus (cut-offs at ≥ 3 mm, ≥ 4 and ≥ 6 mm). Presence of caries was examined with a dental explorer, teeth loss and the reason for each tooth loss as self-reported by the participants were recorded.

### Statistical analysis

Descriptive statistics, mean, standard deviation, percentage and frequency were calculated for all the variables along with tabular presentations. Univariate and forward conditional method for multivariate logistic regression was done to assess the crude and adjusted odds ratio with 95% CI to find out the association between tobacco use and other factors with periodontitis. Level of significance was set at *p* < 0.05. Those variables that fell under *p* < 0.2 at univariate analysis, were considered for multivariate logistic regression. All the data collected data was entered into Microsoft Excel 2007 and converted using the statistical software package SPSS 11.5 (SPSS, Chicago, IL, USA) for further analysis.

## Results

A total of 440 participants with the mean (SD) age of 43.80 (13.17), comprising of 42.3% males and 57.7% females were analyzed in the study. Periodontitis was found to be present in 71.6% (n = 315) of surveyed populations. The average BMI of participants was 24.18 ± 2.74 with the mean PLI score 67.82 ± 21.83 and median BPI with IQR (min–max) score of 43 (23–65) (5–100). The 57 participants who had lost their teeth due to periodontal disease (TLPD), all had some form of periodontitis in their remaining teeth with 57.9% having severe, 33.3% moderate and 8.8% having a mild form of periodontitis. Dental caries were present in 63% of the adults examined. Nearly one in every two individual aged 21–65 years (46.6%) were found to be using some form of tobacco (Table 1).

**Table 1:** Characteristics of study population (n = 440)

Variables	Subcategory	Frequency (%)^a^
Age groups	20–34	110 (25.0)
	35–44	124 (28.2)
	45–65	206 (46.8)
Gender	Females	254 (57.7)
	Males	186 (42.3)
SES	Upper class	16 (3.6)
	Lower middle	166 (37.7)
	Lower	258 (58.6)
Brushing frequency	≤ Once/day	312 (70.9)
	≥ Twice/day	128 (29.1)
Tobacco users	Yes	205 (46.6)
	No	234 (53.2)
Smoking status	Current smokers/Bidi smokers	91 (20.7)
	Non smokers	349 (79.3)
Smokeless tobacco (SLT)	Users	152 (34.5)
	Non users	288 (65.5)
	Khaini users	101 (66.4)
	SLT chewers	51 (33.6)
OHI-S	Good	67 (15.2)
	Fair	202 (45.9)
	Poor	171 (38.9)
Teeth loss	Present	218 (49.5)
Reason for teeth loss	Caries	115 (52.7)
	Periodontitis	57 (26.1)
	Others	46 (21.1)
Periodontitis	Present	315 (71.6)
	Absent	125 (28.4)

^a^Column percentage

(Table 2) The prevalence of periodontitis and its severity were significantly associated with age.

**Table 2:** Distribution of periodontitis and its severity according to age (CDC-AAP case definition) [25]

Periodontitis	Age groups (%)			Total	*p* value
	20–34	35–44	45–65		
Present	47 (42.7)	80 (64.5)	188 (91.3)	315 (71.6)	< 0.001
Absent	63 (57.3)	44 (35.5)	18 (8.7)	125 (28.4)	
Total	110 (100)	124 (100)	206 (100)	440 (100)	
Severity of periodontitis					
Mild	38 (80.9)	23 (28.8)	42 (22.3)	103 (32.7)	< 0.001
Moderate	6 (12.8)	45 (56.2)	61 (32.4)	112 (35.6)	
Severe	3 (6.4)	12 (15.0)	85 (45.2)	100 (31.7)	
Total	47 (100)	80 (100)	188 (100)	315 (100)	

Mild, moderate and severe periodontitis was present in 32.7%, 35.6% and 31.7% respectively in the surveyed population. The PD ≥ 4 mm were present in 181 (57.5%) sites and ≥ 5 mm in 111 (35.2%) sites amongst the periodontitis patients. A total of 26.3% of the population were affected with deep PD in 35–44 years age group that increased to 43.6% in 45–65 years age group.

Tobacco users were significantly associated with periodontitis and its severity (*p* < 0.001).

Among tobacco users, mild, moderate and severe periodontitis was present in 20.0%, 35.4% and 44.6% respectively in the surveyed population (Table 3).

**Table 3:** Association of periodontitis and its severity amongst the tobacco users

Periodontitis	Tobacco users (%)	Non-tobacco users (%)	Total (%)	*p* value
Present	175 (85.4)	140 (59.6)	315 (71.6)	< 0.001
Absent	30 (14.6)	95 (40.4)	125 (28.4)	
Total	205 (100)	235 (100)	440 (100)	
Severity of periodontitis				
Mild	35 (20.0)	68 (48.6)	103 (32.7)	< 0.001
Moderate	62 (35.4)	50 (35.7)	112 (35.6)	
Severe	78 (44.6)	22 (15.7)	100 (31.7)	
Total	175 (100)	140 (100)	315 (100)	

The factors which showed statistically significant association with periodontitis in multivariate analysis were age groups of the participants, smokers, khaini users, plaque accumulation and teeth loss. The table clearly illustrates that periodontitis increase with increasing age. Older age groups had an increased risk of having periodontitis (OR = 7.58 [95% CI 3.93–14.61]), when compared to younger age groups. A statistically significant association was also found between tobacco users (smokers and khaini users) and periodontitis. Logistic regression has shown that when compared to non-smokers, cigarette/bidi smoking had an increased risk for developing periodontitis (OR = 3.14 [95% CI 1.36–7.27]). Similarly, the use of smokeless tobacco in the form of khaini (OR = 2.27 [95% CI 1.12–4.61]) had an increased risk for having periodontitis when compared to adults who never used khaini. Increased prevalence of periodontitis was also reflected in participants with increased tooth mortality, and the association was statistically significant (OR = 2.02 [95% CI 1.21–3.38]). Plaque accumulation also showed a statistically significant association with the prevalence of periodontitis, but, with a low odds ratio (OR = 1.01 [95% CI 1.00–1.02]). The number of males in our study were low, however they had a higher prevalence of periodontal tissue loss (OR = 1.37 [95% CI 0.75–2.48]). Participants with poor oral hygiene, (OR = 1.86 [95% CI = 0.77–4.51]), had increased risk of developing periodontitis when compared to participants with good oral hygiene, but this was not significant at multivariable level. Factors such as the socio-economic status, brushing frequency, and smokeless tobacco chewers did not show a significant association with periodontitis when adjusted for other factors at both univariate and multivariate analysis (Table 4).

**Table 4:** Univariate and multivariate logistic regression analysis between tobacco use and other factors with periodontitis

	Periodontitis present	COR^a^	p value	AOR^b^	95% CI (L-U)	*p* value
Age groups						
20–35	47 (42.7%)	Constant	< 0.001	Constant		
35–44	80 (64.5%)	2.43		1.98	1.12–3.49	< 0.018
45–65	188 (91.3%)	14.00		7.58	3.93–14.61	< 0.001
Gender						
Female	163 (64.2%)	Constant	< 0.001	Constant		
Male	152 (81.7%)	2.49		1.37	0.75–2.48	NS
SES						
Upper	14 (87.5%)	3.08	0.363	–		
Lower middle	122 (73.5%)	1.22				
Lower	179 (69.4%)	Constant				
Brushing frequency						
≤ Once/day	229 (73.4%)	Constant	0.190	Constant		
≥ Twice/day	86 (67.2%)	1.34		1.19	0.68–2.06	NS
OHI-S						
Good	34 (50.7%)	Constant	< 0.001	Constant		
Fair	133 (65.8%)	1.87		1.01	0.50–2.01	NS
Poor	148 (86.5%)	6.24		1.86	0.77–4.51	NS
Missing teeth						
Absent	134 (60.4%)	Constant	< 0.000	Constant		
Present	181 (83.0%)	3.21		2.02	1.21–3.38	0.007
Smoking status						
Absent	232 (66.5%)	Constant	< 0.001	Constant		
Present	83 (91.2%)	5.23		3.14	1.36–7.27	0.007
Khaini						
Absent	226 (66.7%)	Constant	< 0.001	Constant		
Present	89 (88.1%)	3.70		2.27	1.12–4.61	0.023
SLT chewers						
Absent	277 (71.2%)	Constant	0.623	–		
Present	38 (74.5%)	1.18				
BMI	24.6 + 2.6	1.23	< 0.001	1.07	0.96–1.19	NS
PLI	71.9 + 20.2	1.03	< 0.001	1.01	1.00–1.02	0.004
BPI	49.7 + 24.4	1.02	< 0.001	1.00	0.99–1.02	NS

^a^Crude odds ratio

^b^Adjusted odds ratio

## Discussion

In the presented study, the overall prevalence of periodontitis was found to be 71.6%. Subjects of Asian ethnicity are known to have the third-highest prevalence of periodontitis [26]. Surveys conducted in India, Nepal, and Vietnam have also reported, one-third to half of the middle-age population, are affected with periodontitis [27]. However, the case-based definition used for periodontitis varies from study to study, and identifying the true prevalence of periodontitis continues to be a challenge. Despite this, the prevalence of periodontitis is high in the surveyed population and the factors responsible are poor oral hygiene, presence of plaque along with tobacco consumption rather than gender, geography or economic status.

Periodontitis is a multifactorial disease that may be modifiable or non-modifiable [28]. Our study identified tobacco smoking, smokeless tobacco in the form of khaini and other factors such as age, plaque accumulation and teeth loss as significant factors associated with periodontitis. Similar findings have been reported by Bhat et al. in 2018 [29] who concluded that sociodemographic factors such as age, plaque, and tobacco are the main risk indicators to periodontitis in a rural Indian population. Age has been described as a non-modifiable predisposing factor, and with ample of epidemiological evidence [30, 31] suggesting it, this study further adds to evidence that the periodontitis tends to cumulate for life. Plaque is considered the primary etiological factor for periodontitis [32], and it’s also a preventable factor that forms the basis for management of periodontitis. In this study, over 90% of the population answered that they use a toothbrush to clean their teeth at least one time daily. However, plaque accumulation showed significant association with periodontitis in the assessed population. The number of lost teeth in adults has also been used as a marker for periodontitis in the epidemiologic literature [33, 34]. Our study showed a two-fold increase in the trend of teeth loss, and this could be attributed to periodontal reasons (loss of attachment), presence of dental caries and tobacco use.

This study is one of the few cross-sectional surveys conducted to document the impact of smoking and smokeless tobacco in alternative forms on the periodontium in a rural adult Nepalese population. The influence of tobacco smoking has been studied extensively and has been implicated as one of the important risk factors to periodontitis [35, 36]. The prevalence of tobacco smoking was 20.7% in our study and the results are in accordance with the STEPS survey done in 2012–2013 amongst the Nepalese population [37]. An almost two to four-fold increased risk of developing periodontitis is attributable to smoking as compared with adults who never smoke [38–40]. The current study also showed cigarette smokers to have a three-fold increase in risk for periodontitis than non-smokers. There is a growing perception that SLT use is relatively safer than cigarette smoking and may be an alternative to tobacco smoking [41]. In our study, 83.5% of the population using SLT in both forms had periodontitis. Studies conducted in districts of Karnataka, India and NHANES III survey representing the U.S population, reported a nearly two-fold increase in risk for periodontitis among SLT users [15, 42]. Nepal STEPS survey reveals khaini to be the most common SLT product used followed by chewing tobacco [43]. Our study concluded that participants consuming khaini (66.4% of adults in our study consumed SLT in the form of khaini) were almost twice as likely to develop periodontitis compared to the participants who never used khaini. The results of this study are in agreement to a hospital-based study done in India by Katuri et al. in 2016 [44] which showed the most commonly used SLT product as khaini and concluded SLT users to have greater attachment loss. Similarly, studies done by Kulkarni et al. [21] and Kathiriya et al. [45] in 2016 reported gutkha and khaini to be the most commonly used products, and identified SLT to have similar impact on periodontium as tobacco smoke. In countries like India and Nepal, over 90% of SLT users use tobacco as the main constituent or often betel quid, slaked lime, catechu are added to tobacco [46]. Therefore, nicotine exposure may exert a wide range of effects on the periodontal tissues. Traditional khaini available in south east Asian countries has a high pH and nicotine content, that may facilitate rapid absorption of chemicals through oral mucosa making the population more susceptible to periodontitis [11]. Few researchers have also reported a strong association between SLT chewers and periodontal diseases [47, 48]. However, our study, showed no statistically significant association between SLT chewers and periodontitis. The exact reason for the decrease in periodontitis in SLT chewers is unknown; but one explanation could be, that unlike traditional khaini, which is placed in-between the teeth and gums and sucked slowly over time, chewing SLT builds and mixes with the saliva to form a juice, that is either spat out or swallowed, exerting a lesser effect of harmful chemicals on the periodontium [49]. Variations in the effects of SLT products in relation to periodontium exist across the globe and, therefore, the results should be interpreted based on population studied. However, it would be fair to predict that due to pervasive use of SLT, periodontitis will be higher than projected and this study indicates that SLT in the form of khaini had a significant generalized periodontal involvement.

The limitations of the study include that only those participants who attended the dental health camps were examined and thus, it may not be a complete representation of the population. Also, the study faced serious constraints in terms of resources, lack of additional experts and time, as single-day health camps were organized in different wards. Therefore, studies in a large number of populations in the community are needed to further validate the impact of different forms of tobacco products on the periodontium.

## Conclusion

Periodontitis is prevalent in the surveyed Nepalese population. The findings contribute to the evidence of smoking along with smokeless tobacco in the form of khaini as significant factors associated with periodontitis.

## Supplementary Information

**Additional file 1.** Pre-tested semi-structured questionnaire.

## Data Availability

The datasets supporting the findings of this article are available from the corresponding author.

## Abbreviations

AOR: Adjusted odds ratio
BMI: Body mass index
BPI: Bleeding point index
COR: Crudes odds ratio
CAL: Clinical attachment loss
CDC-AAP: Center for disease control-American academy of periodontology
CEJ: Cemento-enamel junction
NS: Not significant
OHI-S: Simplified oral hygiene index
PD: Probing depth
PLI: Plaque index
SES: Socio-economic status
SD: Standard deviation
SLT: Smokeless tobacco
TLPD: Tooth loss due to periodontal disease
UNC-15: University of North Carolina-15
