# Supplementary material for: Current status of periodontitis and its association with tobacco use amongst adult population of Sunsari district, in Nepal
Source: BMC Oral Health. 2021 Feb 12;21:66. doi: 10.1186/s12903-021-01416-3 (PMC7881591; doi:10.1186/s12903-021-01416-3)
Supplement: Supplementary file 1 — Additional file 1. Pre-tested semi-structured questionnaire. [file 12903_2021_1416_MOESM1_ESM.docx]

**Supplementary Information**

Additional file 1: Pre-tested semi-structured questionnaire

**Name:**

**Age: Gender:** 1. Male 2. Female

**Address:** **Contact number:**

**Occupation:**

Profession Semi-Profession Clerical

Shop-owner Farmer Skilled worker

Semi-skilled worker Unskilled worker Unemployed

**Height: Weight:**

**Chief complaint:**

**Medical history:**

**Brushing frequency:**  0 /1 /2 /3

**Brushing method:**  scrub /vertical /combined

**Method of cleaning:** Finger /toothbrush /others

**Brushing material:**  charcoal /toothpastes /others

**Education:**

Professional or Honours Graduate or Post-Graduate

Intermediate or Post-High-School Diploma High School Certificate

Middle School Certificate Primary School or Literate

Illiterate

**Annual income (**Family income per month in rupees)

≥45751 22851 – 45750 17151 – 22850

11451 – 17150 6851 – 11450 2301 – 6850

≤2300

**Smoking status:** Current smoker/ non-smoker **Bidis:**

Based on 20 cigarettes/pack (Grossi et al 1994)

**Pack year**: no. of cig per day X no. of years smoked **/** 20

Light smoker (>0 and ≤4.45 pack year) (1-2734 packs)

Moderate smoker: >4.45 and <15 pack year (2735-7300 packs)

Heavy smoker: >15 pack years (>7300 packs)

**Oral SLT: (since/times/how long) Current chewer/ former chewer/ non chewer**

1. Betelnut quid with tobacco 2. Zarda (*tobacco leaves with slaked lime*)
2. Gutkha (*mixture of powdered tobacco, areca nut, slaked lime, and catechu*)
3. Mawa (*areca nut, tobacco, and slaked lime*) 5. Khaini *(tobacco with slaked lime)*
4. Snuff *(Dry and moist)*  7. Surti leaves 8. Paan masala

**Other forms: (if any)**

Pipe smokers Cigars Electronic cigarettes

Hookah Sulfa Chillum Kankad

No. of Missing teeth (tooth loss due to periodontal causes/other causes):

No. of dental Caries:

No. of filled teeth:

**ORAL HYGIENE STATUS:**

ORAL HYGIENE INDEX SIMPLIFIED (OHI-s):

**CALCULUS INDEX: CI-s SCORE:**

| **16** | **11** | **26** |
| --- | --- | --- |
| **46** | **31** | **36** |

**DEBRIS INDEX: DI-s SCORE=**

| **16** | **11** | **26** |
| --- | --- | --- |
| **46** | **31** | **36** |

**OHI-s SCORE = DI-s + CI-s = -------------------------**

**PLAQUE CONTROL RECORD (PLI):**

**18 17 16 15 14 13 12 11 21 22 23 24 25 26 27 28**

|  |  |  |  |  |  |  |  |  |  |  |  |  |  |  |  |  |  |  |  |  |  |  |  |  |  |  |  |  |  |  |  |  |  |  |  |  |  |  |  |  |  |  |  |  |  |  |  |
| --- | --- | --- | --- | --- | --- | --- | --- | --- | --- | --- | --- | --- | --- | --- | --- | --- | --- | --- | --- | --- | --- | --- | --- | --- | --- | --- | --- | --- | --- | --- | --- | --- | --- | --- | --- | --- | --- | --- | --- | --- | --- | --- | --- | --- | --- | --- | --- |
|  | | |  | | |  | | |  | | |  | | |  | | |  | | |  | | |  | | |  | | |  | | |  | | |  | | |  | | |  | | |  | | |

**48 47 46 45 44 43 42 41 31 32 33 34 35 36 37 38**

|  |  |  |  |  |  |  |  |  |  |  |  |  |  |  |  |  |  |  |  |  |  |  |  |  |  |  |  |  |  |  |  |  |  |  |  |  |  |  |  |  |  |  |  |  |  |  |  |
| --- | --- | --- | --- | --- | --- | --- | --- | --- | --- | --- | --- | --- | --- | --- | --- | --- | --- | --- | --- | --- | --- | --- | --- | --- | --- | --- | --- | --- | --- | --- | --- | --- | --- | --- | --- | --- | --- | --- | --- | --- | --- | --- | --- | --- | --- | --- | --- |
|  | | |  | | |  | | |  | | |  | | |  | | |  | | |  | | |  | | |  | | |  | | |  | | |  | | |  | | |  | | |  | | |

**% of surfaces with plaque:**

**ASSESSMENT OF BLEEDING COMPONENT:**

**BLEEDING POINT INDEX (BPI): FACIAL**

**18 17 16 15 14 13 12 11 21 22 23 24 25 26 27 28**

|  |  |  |  |  |  |  |  |  |  |  |  |  |  |  |  |
| --- | --- | --- | --- | --- | --- | --- | --- | --- | --- | --- | --- | --- | --- | --- | --- |
|  |  |  |  |  |  |  |  |  |  |  |  |  |  |  |  |

**48 47 46 45 44 43 42 41 31 32 33 34 35 36 37 38**

**% of Bleeding Points:**

**PERIODONTAL STATUS:**

**ASSESSMENT OF POCKET DEPTH (PROBING DEPTHS):**

**FACIAL**

**18 17 16 15 14 13 12 11 21 22 23 24 25 26 27 28**

|  |  |  |  |  |  |  |  |  |  |  |  |  |  |  |  |
| --- | --- | --- | --- | --- | --- | --- | --- | --- | --- | --- | --- | --- | --- | --- | --- |
|  |  |  |  |  |  |  |  |  |  |  |  |  |  |  |  |

**48 47 46 45 44 43 42 41 31 32 33 34 35 36 37 38 LINGUAL**

**ASSESSMENT OF CLINICAL ATTACHMENT LOSS: FACIAL**

**18 17 16 15 14 13 12 11 21 22 23 24 25 26 27 28**

|  |  |  |  |  |  |  |  |  |  |  |  |  |  |  |  |
| --- | --- | --- | --- | --- | --- | --- | --- | --- | --- | --- | --- | --- | --- | --- | --- |
|  |  |  |  |  |  |  |  |  |  |  |  |  |  |  |  |

**48 47 46 45 44 43 42 41 31 32 33 34 35 36 37 38**
